# Inactivating TDP2 missense mutation in siblings with congenital abnormalities reminiscent of fanconi anemia

**DOI:** 10.1007/s00439-023-02589-3

**Published:** 2023-08-10

**Authors:** Guido Zagnoli-Vieira, Jan Brazina, Kris Van Den Bogaert, Wim Huybrechts, Guy Molenaers, Keith W. Caldecott, Hilde Van Esch

**Affiliations:** 1 Genome Damage and Stability Centre, School of Life Sciences, University of Sussex, Falmer, Brighton, BN1 9RQ UK; 2 Present Address: Wellcome Trust Cancer Research UK Gurdon Institute, Tennis Court Road, Cambridge, CB2 1QN UK; 3 Center for Human Genetics, University Hospitals Leuven, Herestraat 49, 3000 Louvain, Belgium; 4 Pediatric Orthopedics, Department of Orthopedics, University Hospitals Leuven, Herestraat 49, 3000 Louvain, Belgium

## Abstract

Mutations in *TDP2*, encoding tyrosyl-DNA phosphodiesterase 2, have been associated with a syndromal form of autosomal recessive spinocerebellar ataxia, type 23 (SCAR23). This is a very rare and progressive neurodegenerative disorder described in only nine patients to date, and caused by splice site or nonsense mutations that result in greatly reduced or absent TDP2 protein. TDP2 is required for the rapid repair of DNA double-strand breaks induced by abortive DNA topoisomerase II (TOP2) activity, important for genetic stability in post-mitotic cells such as neurons. Here, we describe a sibship that is homozygous for the first TDP2 missense mutation (p.Glu152Lys) and which presents with clinical features overlapping both SCAR23 and Fanconi anemia (FA). We show that in contrast to previously reported SCAR23 patients, fibroblasts derived from the current patient retain significant levels of TDP2 protein. However, this protein is catalytically inactive, resulting in reduced rates of repair of TOP2-induced DNA double-strand breaks and cellular hypersensitivity to the TOP2 poison, etoposide. The TDP2-mutated patient-derived fibroblasts do not display increased chromosome breakage following treatment with DNA crosslinking agents, but both TDP2-mutated and FA cells exhibit increased chromosome breakage in response to etoposide. This suggests that the FA pathway is required in response to TOP2-induced DNA lesions, providing a possible explanation for the clinical overlap between FA and the current TDP2-mutated patients. When reviewing the relatively small number of patients with SCAR23 that have been reported, it is clear that the phenotype of such patients can extend beyond neurological features, indicating that the TDP2 protein influences not only neural homeostasis but also other tissues as well.

## Introduction

Mutations in *TDP2*, encoding tyrosyl-DNA phosphodiesterase 2, have been associated with intellectual disability, cerebellar ataxia, and seizures; a disease known as autosomal recessive spinocerebellar ataxia, type 23 (SCAR23, OMIM #616949) (Gómez-Herreros et al. 2014; Zagnoli-Vieira et al. 2018; Ciaccio et al. 2019; Errichiello et al. 2020; Zoghi et al. 2021). This is a very rare and progressive neurodegenerative disorder described in only nine patients to date, and caused by splice site or nonsense mutations that result in greatly reduced or absent TDP2 protein. Seizure onset can occur as early as the first months of life, but can also occur late in puberty, often preceding the ataxia. Brain imaging is often not informative, although cerebellar atrophy was reported in one patient (Ciaccio et al. 2019). In most patients, mild intellectual disability, developmental delay, and speech problems are also present.

TDP2 (tyrosyl-DNA phosphodiesterase 2) is a highly conserved member of the endonuclease/exonuclease/phosphatase (EEP) family of enzymes (Ledesma et al. 2009). TDP2 is required for the rapid repair of DNA double-strand breaks (DSBs) induced by abortive DNA topoisomerase II (TOP2) activity, by removing covalently trapped TOP2 from the DSBs and thereby allowing their direct ligation during non-homologous end-joining (NHEJ) (Zeng et al. 2011; Gómez-Herreros et al. 2013). This provides an error-free mechanism for NHEJ-mediated DSB repair, which may be particularly important for genetic stability and transcriptional proficiency in post-mitotic cells such as neurons, in which other sources of error-free DSB repair are likely absent (Caldecott 2012; Gómez-Herreros et al. 2014, 2017).

Here, we describe a sibship that is homozygous for the first *TDP2* missense mutation (E152K) (SNP rs754324675) identified in human neurological disease, and which presents with clinical features overlapping both SCAR23 and Fanconi anemia. Fanconi anemia (FA) is a rare recessive DNA-repair disease that may lead to bone marrow failure (aplastic anemia), leukemia, and/or solid tumors. In addition, specific skeletal anomalies are often present (radial ray defects) as well as developmental delay and congenital urogenital and/or heart defects.

Here, we show that in contrast to previously reported SCAR23 patients, fibroblasts derived from the current patients retain significant levels of TDP2 protein. This protein is catalytically inactive, however, resulting in reduced rates of repair of TOP2-induced DNA double-strand breaks and cellular hypersensitivity to the TOP2 poison, etoposide. Despite the phenotypic overlap with FA, the TDP2-mutated patient-derived fibroblasts do not display increased chromosome breakage following treatment with DNA crosslinking agents. Notably, however, patient-derived fibroblasts from both TDP2-mutated and FA patients exhibit increased chromosome breakage in response to etoposide. This suggests that the FA pathway is required in response to TOP2-induced DNA lesions, providing a possible explanation for the clinical overlap between FA and the current TDP2-mutated patients.

## Methods

### Genomic variant diagnosis

Genomic DNA was extracted from peripheral blood via standard protocols. Molecular karyotyping was conducted using OGT Cytosure 180 k, v3 array. The clinical exome analysis was done using the Nimblegen V4 Panel clinical exome, consisting of 6178 genes. We analyzed the two affected siblings (ii-2 and ii-3) and their parents and data were obtained with massive parallel sequencing on Illumina Hiseq2500. Annotation of the variants was done using Genome build GRCh37, RefSeq78 and Cartagenia Bench Lab NGS (version 5.0). We filtered sequence variants in a stepwise manner to exclude synonymous variants, non-exonic SNVs, indels and variants with a minor allele frequency > 1% in gnomAD (version v2.1.1), the 1000 Genomes Project, and internal exome databases. Using the parental data, we looked for “de novo”, X-linked as well as combined heterozygous and homozygous mutations in the affected siblings. Variants were confirmed by Sanger sequencing in the siblings as well as in the parents.

### Chromosomal breakage assays using etoposide or diepoxybutane

Epstein Barr Virus-transformed peripheral-blood lymphocytes (EBV-PBLs) of the two affected siblings, the healthy brother (no carrier), a healthy control line, as well as two established Fanconi cell lines (F1 and F2) were used to study chromosomal breakage. Fanconi patient F1 carries a homozygote pathogenic class 5 variant in *FANCG* (NM_004629.1), namely c.637_643delTACCGCC (p.Tyr213Lysfs*6). Fanconi patient F2 is combined heterozygote for two mutations in *FANCC* (NM_000136.2) namely c.520C > T (p.Arg174*) and c.455dupA (p.Asn152Lysfs*9), both class 5 mutations. Peripheral blood lymphocytes were cultured according to standard procedures. After 24 h of incubation, 20 nM Etoposide (Sigma E1383) was added to the culture for 27 h. After 24 h, cells were arrested in metaphase through a 3 h treatment with 10 μg/ml KaryoMAX Colcemid solution (Gibco), treated with 0.075 M KCl, fixed in methanol:acetic acid (3:1), spread onto glass slides and air-dried. Slides were stained with bisBenzimide H 33258 (Sigma), exposed to UV light and finally stained with Giemsa. For DEB analysis, the procedure is similar. Instead of etoposide, a 0.004% diepoxybutane solution was added to the cultures after 24 h of incubation. In both cases, a minimum of 25 cells were analyzed and the number of breakages per mitosis was calculated.

### Cell culture

Lymphoblastoid cells from unaffected and affected individuals were grown in RPMI 1640 medium (Gibco) at 10% FCS in the presence of penicillin (10U/mL), streptomycin (10 μg/mL) and 2 mM l-glutamine. Primary fibroblasts from affected individual ii-3 were derived from skin biopsy and grown in MEM (Gibco) at 20% FCS in the presence of penicillin (10U/mL), streptomycin (10 μg/mL) and 2 mM l-glutamine. Fibroblasts and lymphoblasts from previously characterized SCAR23 (TDP2-mutated) patients (850BR, IV-9, IV-14 and IV-16) and controls (1BR, IV-2) were described previously described (Gómez-Herreros et al. 2014; Zagnoli-Vieira et al. 2018). RPE-1 hTERT cas9 cells and RPE-1 h-TERT Cas9 *TDP2*^*−/−*^ cells were cultured in DMEM:F12 mix (Gibco) at 10% FCS in the presence of penicillin (10U/mL), streptomycin (10 μg/mL) and 2 mM l-Glutamine. All cells were maintained at 37 °C and 5% CO_2_.

### TDP2^−/−^ RPE-1 hTERT Cas9 cells and mCherry-TDP2/mCherry-TDP2^E152K^ expression

RPE-1 hTERT Cas9 cells were a gift from Prof. Steve Jackson, and have been described previously (Balmus et al. 2019). RPE-1 hTERT Cas 9 *TDP2*^*−/−*^ were created by transfecting the sgRNA 5′-CCTGTAGAAATATCACATCT-3′ that targets TDP2 exon 4 into RPE-1 hTERT Cas9 cells. Following clonal selection, gene targeting was confirmed by western blotting. For complementation studies, the human TDP2 ORF was cloned into the *Hpa*I/*Afe*I sites of the Vector Builder mCherry plamid VB200726-1045daz, using Gibson assembly (NEB) with the indicated forward (5′-AGGATGACGATGACAAGAGCATGGAGTTGGGGAGTTGCCT-3′) and reverse primers (5′-TCGAGGTCGACACGCGTGTTCAATATTATATCTAAGTTGCACAGAAGACC-3′), creating mCherry-TDP2. mCherry-TDP2^E152K^ was generated by site-directed mutagenesis (Q5 Site-directed mutagenesis kit; NEB) using mCherry-TDP2 and the forward/reverse primers 5′-CAGATGTGATATTTCTACAGaAAGTTATTCCCCCATATTA-3′ & 5′-TAATATGGGGGAATAACTTtCTGTAGAAATATCACATCTGGGCT-3′. The final constructs were packaged using psPAX2 (Addgene #12260) and pMD2.G (Addgene #12259) and lentiviral particles transduced into cells for 24 h prior to selection with 1 mg/mL Geneticin (Gibco) for 7 days.

### Western blotting

Protein samples were prepared in Laemmli sample buffer (2% SDS, 10% glycerol, 60 mM Tris–HCl pH6.8) and heated for 10 min at 95 °C. Sample proteins were quantified using Bicinchoninic acid assay (BCA) reagent (Pierce) according to manufacturer instructions. Prior to loading, samples were supplemented with 100 mM dithiothreitol and 0.005% bromophenol blue. Following SDS-PAGE electrophoresis and western blotting, TDP2 and KU80 were detected using a rabbit anti-TDP2 primary antibody (Thomson et al. 2013), and KU80 was detected as a loading control using an anti-Ku80 rabbit monoclonal primary antibody (Abcam Ab80592). For overexpressed mCherry-tagged TDP2, anti-mCherry antibody (Abcam ab167453) was employed.

### Tyrosyl DNA phosphodiesterase (TDP) assays

TDP assays were performed as previously described (Zagnoli-Vieira et al. 2018). In brief, cell extracts were prepared by resuspension of lymphoblastoid cells in lysis buffer (40 mM Tris/HCl pH 7.5, 100 mM NaCl, 0.1% Tween-20, 1 mM DTT, 1 mM PMSF, 1 × EDTA free protease cocktail inhibitor), followed by 30 min incubation on ice and mild sonication using a BioRuptor (Diagenode) for five cycles of 30 s on/off. The cell extract was then clarified by centrifugation for 10 min at 4 °C at 16,000 × *g* in a microfuge and the protein concentration quantified using the bicinchoninic acid (BCA) assay reagent (ThermoFisher). 3 μg of clarified cell extract was incubated with 40 nM of TDP2 substrate (Cy5-5′Tyrosine-ssDNA_19_-BHQ) or TDP1 substrate (BHQ-ssDNA_13_-3′Tyrosine-Cy5) diluted in reaction buffer (50 mM Tris/HCl pH8.0, 10 mM MgCl_2_, 80 mM KCl, and 1 mM DTT, 0.05% Tween-20, 6 μM unlabeled 19 bp oligo) in a total volume of 6 µl at room temperature for 45 min. Cy5 fluorescence was measured at 650 nm on a BMG PHERAstar plate reader. Benzonase (Sigma) was used as positive control.

### Immunoflourescence

Cells were grown in 24-well plates (Greiner SensoPlate) until confluent and then treated for 30 min with 50 µM etoposide or irradiated with γ-irradiation (2 Gy). After treatment, cells were rinsed with PBS and replenished with fresh media or fixed for 10 min in PBS containing 4% paraformaldehyde at the indicated time points. Following fixation, cells were permeabilised (20 min, 0.2% Triton X-100 in PBS), blocked (1 h in PBS-5% BSA), and incubated with anti-γH2AX (Millipore, 05–636, 1:2500) and anti-CENP-F (Abcam, ab5, 1:2500) antibodies for 3 h in PBS containing 5% BSA. Cells were then washed (3 × 5 min in PBS containing 0.1% Tween-20), incubated for 1 h with the corresponding Alexa Fluor conjugated secondary antibody (1:1000, 5% BSA), and washed again as described earlier. Finally, cells were counterstained with DAPI (Sigma, Gillingham, UK) and imaged on Opera Phenix microscope (PerkinElmer) with 40X water immersion objectives. Image analysis and evaluation was done using Harmony High-Content Imaging and analysis software. Cells were gated to the G1 population according to the CENPF signal.

### Clonogenic survival assays

Patient-derived fibroblasts were plated onto feeder layers and 3 h later treated with the indicated concentrations of etoposide or ionising radiation prior to incubation for 21 days to allow the formation of macroscopic colonies. For feeder layers, 1BR cells were irradiated (35 Gy) and plated 24 h before use at 5 × 10^4^ cells/10 cm dish. For RPE-1 hTERT Cas9 cells, clonogenics were performed as above but the feeder layer was omitted, and macroscopic colonies were allowed to form for 10 days. Following colony formation, cells were fixed in 100% ethanol and dishes rinsed with PBS prior to staining with 70% ethanol/1% crystal violet. Dishes were allowed to dry, prior to scoring colonies of > 50 cells. The surviving fraction at each dose was calculated by dividing the average number of colonies in treated dishes by the average number in untreated dishes.

## Results

### Case description

**Fig. 1:**
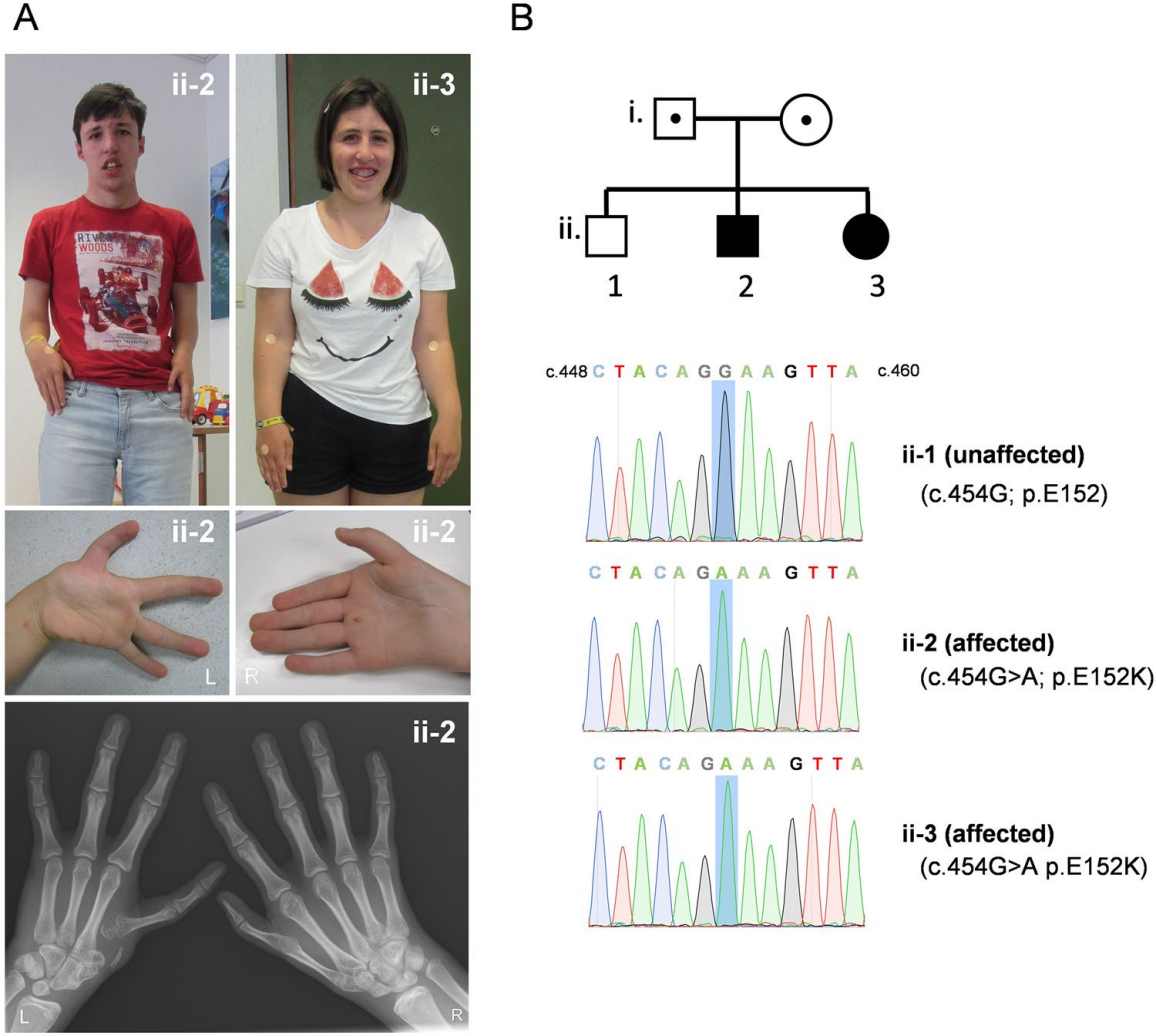
Homozygous missense mutations in *TDP2* in a sibship with skeletal features overlapping Fanconi anemia (FA)-like pathology. **A** Clinical photos of the brother (ii-2) and sister (ii-3) showing the short stature, facial dysmorphism, shorter arms and congenital radial ray anomalies. **B** Pedigree and Sanger sequencing tracing of unaffected (ii-1) and two affected (ii-2 and ii-3) siblings. The position of c.454G and its mutation (c.454G > A; p.E152K) in the affected siblings is indicated by a blue box

The brother (ii-2) and sister (ii-3) are the second and third child of healthy unrelated parents of Caucasian origin (Fig. 1A). The eldest brother (ii-1) is healthy and the family history is negative. The affected siblings were assessed clinically from young age because of developmental delay and congenital malformations. The boy was born after a pregnancy complicated by intrauterine growth retardation. His birthweight was 2.6 kg (p25), length 47.5 cm (p50) and head circumference 32.8 cm (p3) at 36 weeks post-menstrual age. At birth, shorter arms with a bilateral humero-radial synostosis were noted as well as bilateral hypoplastic thumbs (first metacarpal) and radius, more pronounced at the left side (Fig. 1A). Because of suspicion of Fanconi anemia, chromosome breakage using DEB was examined at the age of 1 year, but was negative. Also, standard chromosomal analysis was normal. During further follow-up, a developmental delay was noted for which he attended a special school. As a young child, he experienced some episodes of febrile seizures. At the age of 19 years, he developed partial complex seizures that responded well to medication. Now at the age of 23 years, he has a short stature of 158 cm (− 3.3SD) and a head circumference of 55 cm. He has small dysplastic ears with absent lobuli and small ear canals as well as small deep-set eyes, longer philtrum and hypotonic mouth. He underwent surgery for dysplastic hips and genua valga and developed a minimal scoliosis. He has a moderate intellectual deficit (total IQ 47) and since the age of 21 years his gait is becoming ataxic. The younger sister (ii-3) was born at term with normal birth parameters. She had one episode of generalized epilepsy at the age of 6 months and remained seizure-free under medication. Her development was slow, albeit better than her brother. She has a bilateral dorsal subluxation of the radial head, shorter arms as well as dysplastic hips. Now at the age of 20 years, her final height is 150 cm (− 2.7 SD) and head circumference is 51 cm (− 2.2 SD). She is moderately delayed with a total IQ of 51 and to date, we did not observe ataxia. In the past, a metabolic work-up in both siblings was negative. Ultrasound of the heart and abdomen were also normal. Array comparative genome hybridization on DNA of both siblings showed normal results. Clinical exome analysis in quadruple showed the presence of a homozygous missense mutation c.454G > A (p.Glu152Lys; TDP2^E152K^) in the tyrosyl-DNA phosphodiesterase 2 (*TDP2*) gene (transcript NM_016614.2). Both parents are heterozygous carriers of the variant, and this was confirmed by Sanger sequencing (Fig. 1B). The eldest brother (ii-1) does not carry the variant (Fig. 1B top panel). E152 is a highly conserved residue and the variant is predicted to be damaging by various prediction software. The variant was seen once in the heterozygous state in gnomADv3 database and is absent in ClinVar. No other pathogenic variants (class 4–5) were detected in the exome of both sibs.

### TDP2 protein levels and activity

**Fig. 2:**
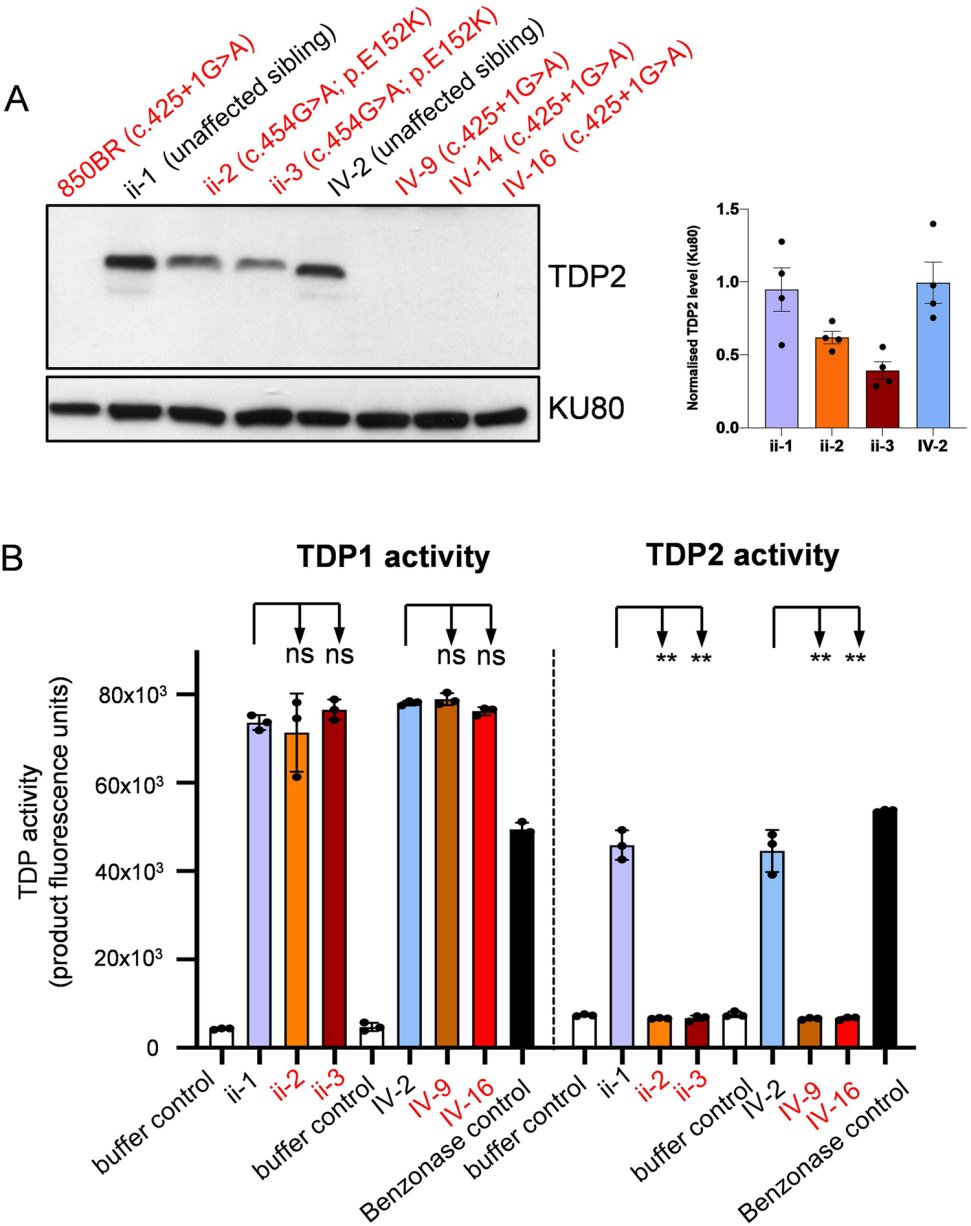
TDP2 protein levels in patients with the TDP2 missense or splice site mutations. **A** Western blot showing levels of TDP2 protein in lymphoblastoid cells (LCls) derived from patients harboring either the homozygous TDP2 missense mutation c.454G > A (p.E152K) (ii-2 and ii-3), the previously reported homozygous TDP2 splicing mutation c.425 + 1G > A (IV-9, IV-14, IV-16) (Gómez-Herreros et al. 2014; Zagnoli-Vieira et al. 2018), or the indicated unaffected sibling controls (i-1, IV-2, respectively). Quantification of four experiments at right panel. **B** Levels of TDP1 and TDP2 activity, measured as previously described (Zagnoli-Vieira et al. 2018) as levels of fluorescent oligonucleotide product of 3′-tyrosyl and 5′-tyrosyl phosphodiesterase activity, respectively, in cell extracts prepared form the indicated LCLs. Data are the mean of three independent experiments and statistically significant differences were determined by one-way ANOVA with Sidak’s multiple comparison test (*ns* not significant; **p ≤ 0.01)

To date, all individuals identified with *TDP2* mutations exhibit a similar pathology of intellectual disability, seizures, and ataxia; a disease now denoted spinocerebellar ataxia autosomal recessive 23 (SCAR23) (Gómez-Herreros et al. 2014; Zagnoli-Vieira et al. 2018; Ciaccio et al. 2019; Errichiello et al. 2020; Zoghi et al. 2021). Table 1 provides an overview of the patients reported to date with molecular and clinical details. However, clinical phenotypes reminiscent of Fanconi anemia, such as radial ray defects, have not previously been reported, suggesting that the molecular defect underpinning the current affected individuals is different. To address this, we first compared cell lines from the unaffected sibling (ii-1) and the two affected siblings (ii-2, ii,3) for levels of TDP2 protein (Fig. 2A). Surprisingly, whereas previously described patients lack detectable TDP2 protein, lymphoblastoid cells from the current patients exhibited significant amounts of residual TDP2 (Fig. 2A). Despite this, tyrosyl phosphodiesterase assays revealed that lymphoblastoid cell extracts from both TDP2^E152K^ patients (ii-2 and ii-3) lacked detectable TDP2 activity (Fig. 2B). This was similar to lymphoblastoid cell extracts prepared from TDP2 patients that we have described previously (IV-9, IV-16) (Gómez-Herreros et al. 2014), in which a donor splice site mutation (c.425 + 1G > A) prevents expression of most if not all TDP2 (Fig. 2B). In contrast, the level of TDP1 activity in each of the patient-derived cell extracts was similar to that in unaffected controls, ii-1 and IV-2 (Fig. 2B). These data indicate that the E152K mutation inactivates the catalytic activity of TDP2; a conclusion consistent with this residue being a key catalytic residue, required for Mg^2+^ coordination (Ledesma et al. 2009; Schellenberg et al. 2016).

**Table 1:** Summary of SCAR23 cases currently reported in the literature including our cases

	(Gómez-Herreros, 2014), IV-9	(Gómez-Herreros, 2014), IV-14	(Gómez-Herreros, 2014), IV-16	(Zagnoli-Vieira 2018)	(Ciaccio, 2019)	(Errichiello, 2020), III-38	(Errichiello, 2020), III-40	(Zoghi, 2021)	Current study, ii-2	Current study, ii-3
Age (years)	32	26	23	6	11	45	39	3	23	19
Sex	Male	Male	Male	Male	Female	Male	Female	Male	Male	Female
Homozygous TDP2 variant	c.425 + 1G > A (intron 3)			c.425 + 1G > A (intron 3)	c.400 > T, p.Arg134Ter	c.636 + 3_636 + 6del (intron 5)		c.4G > T, pGlu2*	c.454G > A, p.E152K	
Seizures/Epilepsy (age of onset)	SGE—tonic (< 2 months)	SGE—tonic (~ 12 years)	SGE—tonic (< 6 months)	Yes (< 5 months)	Focal tonic (12 years)	Focal epilepsy (15 years)	Focal epilepsy (15 years)	No	Febrile/Partial complex seizures (childhood)	Generalized epilepsy at 6 moths. Currently seizure-free under medication
Ataxia	Yes	Yes	Yes	Yes	Yes	Progressive ataxia	Progressive ataxia	Yes	Yes, from 21 years	No
Intellectual disability	Moderate to severe	Moderate to severe	Moderate to severe	Yes	NI	Severe delayed speech development		Severe developmental delay	Developmental delay/moderate ID	Developmental delay/moderate ID
Facial Dysmorphia	Brachycephaly, large ears, hypotelorism, down-slanting palpebral fissures, deep-set eyes, short philtrum, small mouth with high palate, prominent lower lip			Microcephaly	Microcephaly	Broad nasal bridge, low hanging columella, broad interalar distance, bulbous long nose		Narrow palate, protruding columella, short philtrum	Deep set eyes, prominent nasal bridge, hypoplastic earlobes. Microcephaly (ii-3)	
Other Neurological Symptoms/MRI findings	WCB Hypotonia	WCB	WCB	Hypotonia	Dizziness	Hypotonia	Hypotonia	Hypotonia Oculo-motor apraxia	Hypotonia	Hypotonia
					Cerebellar atrophy, mild supratentorial atrophy	Increased hippocampal FLAIR signal intensity	Urinary incontinence			
Others				Neutropenia	Low weight	Short stature	Short stature		Short stature, short arms, dysplastic hips and ears, ears lacking lobuli, small deep-set eyes, bilateral humeroradial synostosis, bilateral hypoplastic thumbs and radius	Short stature, short arms, dysplastic hips, bilateral subluxation of radial head
				Cardiac arrhythmia	Fatigue	Constipation	Low weight			
				Hyponatremia		Hyperextensible joints	Constipation			
				Fatigue		Elbow contractures	Hyperextensible joints			
						Overeating				

Summary of currently known SCAR23 cases. *SGE* symptomatic generalized epilepsy, *WCB* wheelchair bound, *NI* information not available, *AED* anti-epileptic drugs. Adapted from (Gómez-Herreros et al 2014; Zagnoli-Vieira et al 2018; Ciaccio et al 2019; Errichiello et al 2020; Zoghi et al 2021)

### DSB repair proficiency and cellular hypersensitivity to TOP2-induced DSBs

**Fig. 3:**
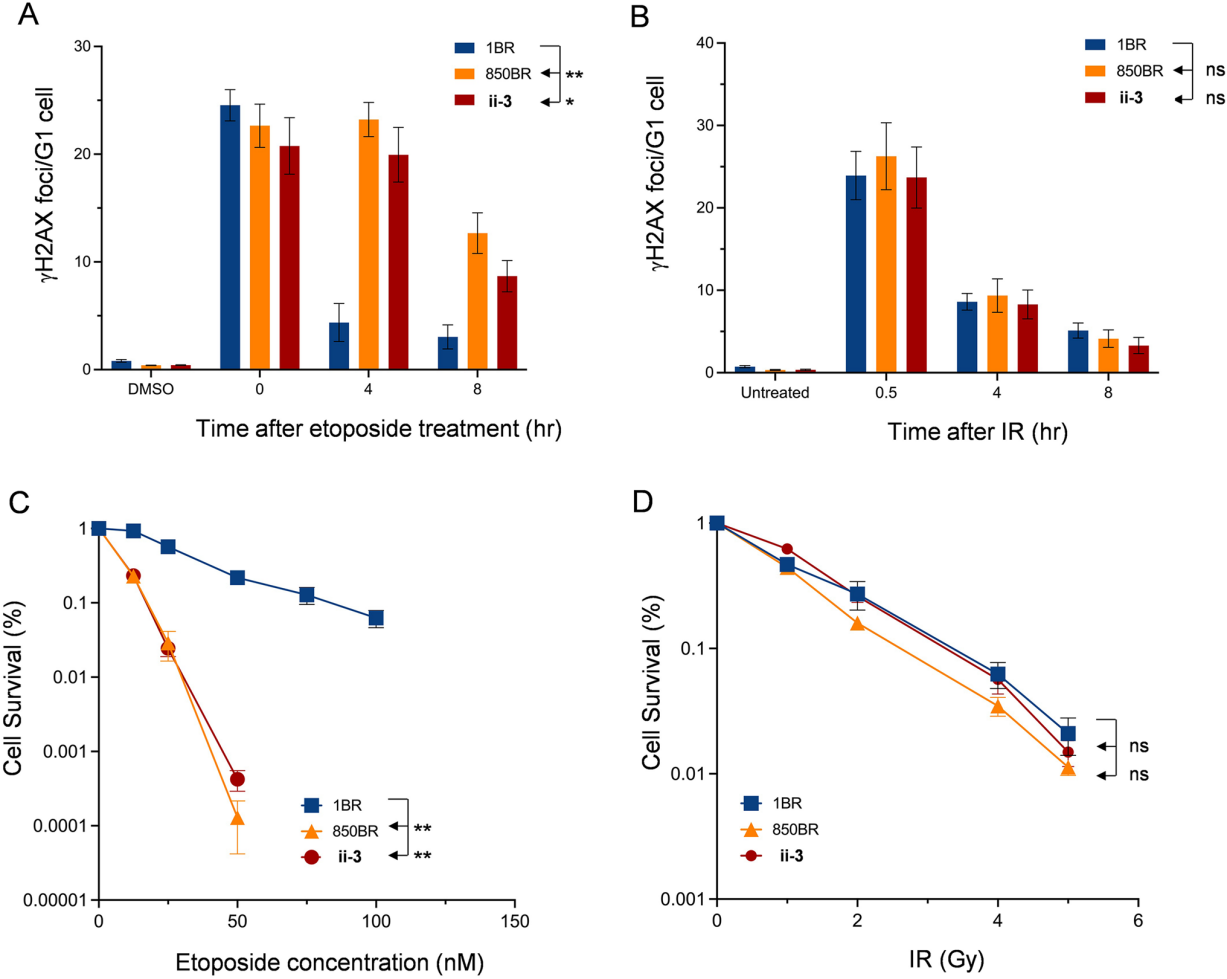
Rates of DSB repair and hypersensitivity of TDP2 patient-derived primary fibroblasts to etoposide. **A**, **B** DSBs were measured by γH2AX immunostaining TDP2 patient-derived primary fibroblasts (850-BR, ii-3) and in primary fibroblasts from an unrelated normal control (1BR) before and after treatment with 50 μM etoposide for 30 min (panel A) or 2 Gy X-rays (panel B), followed by incubation in drug-free medium for the indicated repair periods. Data are the mean of four independent experiments and statistically significant differences were determined by two-way ANOVA with Dunnett’s multiple comparison tests. **C**, **D** Clonogenic survival of the indicated normal and TDP2 patient-derived fibroblasts following treatment with the indicated concentrations/doses of etoposide (panel C) or X-rays (panel D). Data are the mean (± SEM) of 3 independent experiments and statistically significant differences were determined by 2-way ANOVA

TDP2 facilitates the repair of DSBs induced specifically by TOP2, and TDP2 patient-derived cells exhibit significantly reduced rates of TOP2-induced DSB repair (Gómez-Herreros et al. 2013, 2014; Zagnoli-Vieira et al. 2018; Errichiello et al. 2020). To see if this was true in TDP2^E152K^ patient-derived cells we quantified the number of γH2AX foci, a robust measure of DSBs in G1 cells, following treatment with the TOP2 poison etoposide or X-irradiation as a control. Indeed, whereas unrelated unaffected control fibroblasts (1BR) repaired most of the TOP2-induced DSBs within 4 h, TDP2^E152K^ patient-derived fibroblasts (ii-3) repaired far fewer over this period, and retained high levels of TOP2-induced DSBs even 8 h after treatment (Fig. 3A). This defect was similar to that observed in 850BR cells derived from a patient with the *TDP2* splice site mutation (c.425 + 1G > A) in which TDP2 protein is below detectable levels or absent (Fig. 3A). In contrast, both TDP2 patient-derived cell lines repaired DSBs induced by γ-irradiation at normal rates (Fig. 3B).

**Fig. 4:**
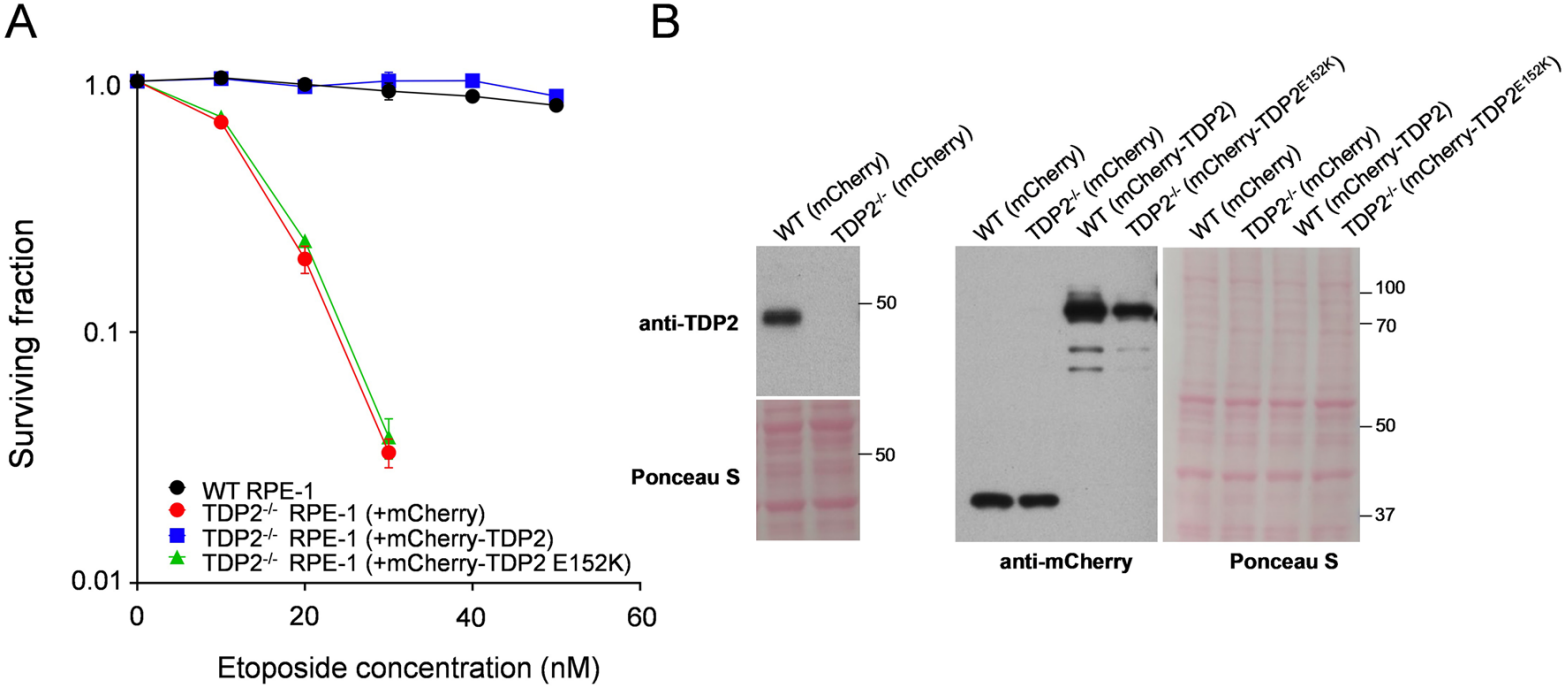
Hypersensitivity of human TDP2-/- RPE-1 cells to etoposide and their complementation by wild-type TDP2 or TDP2 E152K. **A** Clonogenic survival of wild-type RPE-1 cells and *TDP2*^*−/−*^ RPE-1 cells harboring either empty mCherry expression vector, mCherry-TDP2 or mCherry-TDP2^E152K^ following treatment with the indicated concentrations of etoposide. Data are the mean (± SEM) of four independent experiments. **B** Western blot showing levels of endogenous TDP2 (*left)* and mCherry-tagged TDP2 or TDP2^E152K^ (*right*) in the indicated mCherry-expressing cell lines

To examine whether the DSB repair defect imposed by TDP2^E152K^ mutation resulted in hypersensitivity to TOP2-induced DSBs, we examined the clonogenic sensitivity of the patient-derived cells to etoposide. ii-3 cells exhibited a level of hypersensitivity similar to that observed in 850BR cells, when compared to unaffected 1BR control cells (Fig. 3C). In contrast, both of the TDP2 patient-derived cell lines exhibited normal levels of sensitivity to ionising-irradiation (Fig. 3D). To confirm directly that the E152K mutation resulted in etoposide hypersensitivity, we compared expression constructs encoding wild type or mutant TDP2^E152K^ for ability to restore etoposide resistance in *TDP2*^*−/−*^ RPE-1 cells. Indeed, whereas wild-type mCherry-tagged protein fully restored cellular resistance to etoposide, mCherry-tagged TDP2^E152K^ was unable to do so (Fig. 4).

### Chromosome breakage

**Fig. 5:**
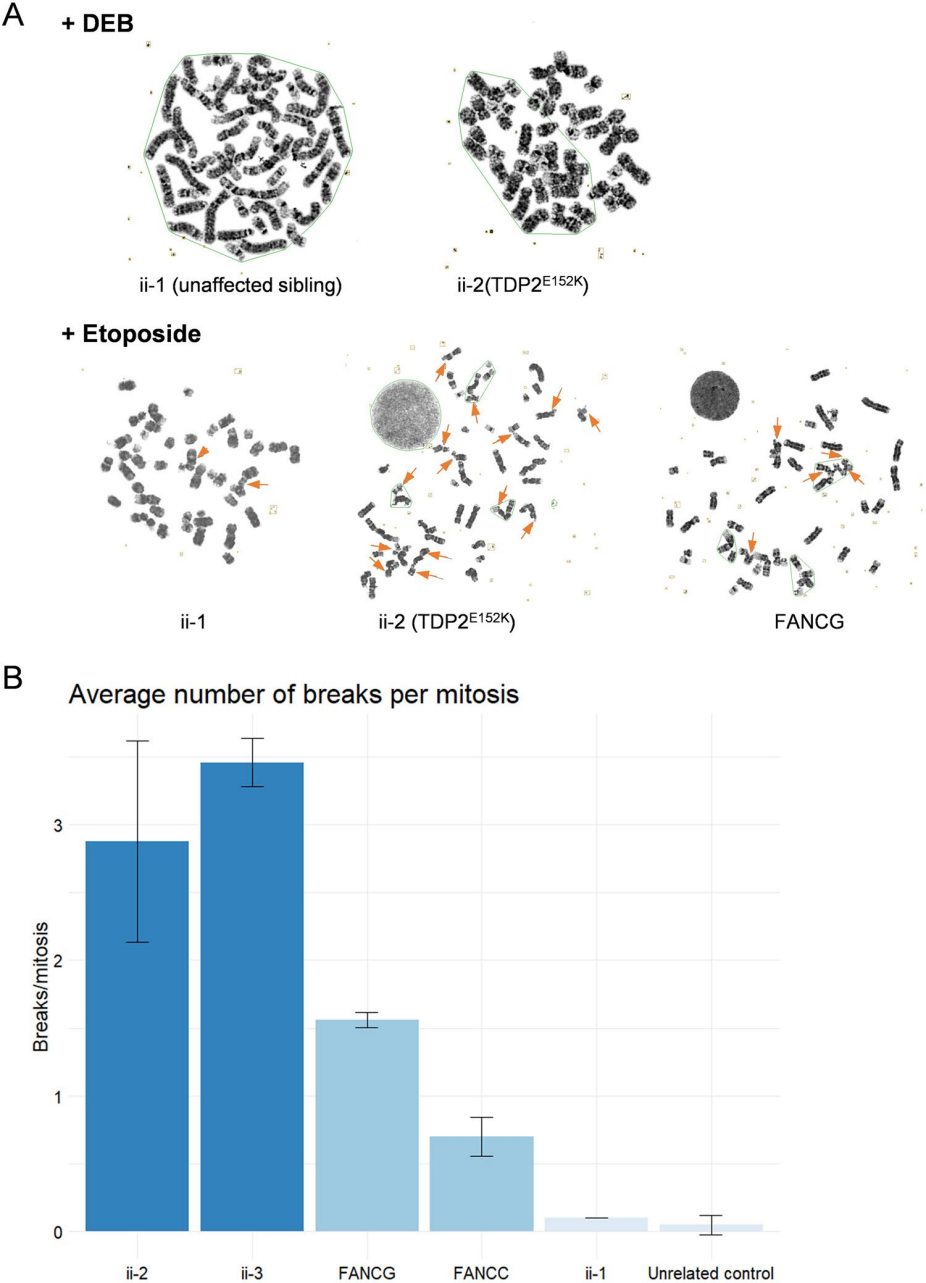
Increased chromosomal breakage following treatment with etoposide. **A** Representative images of patient-derived lymphoblastoid cells, control lymphoblastoid cells and Fanconi derived lymphoblastoid cells, treated with either DEB (upper part) or etoposide lower part. Arrows indicate the presence of a chromosome break. **B** Average number of breaks per mitosis in the different lymphoblastoid cells after treatment with etoposide. Data are the mean of two independent experiments. Plot generated by GGPlot2

Because of the skeletal abnormalities in the proband, which overlap with Fanconi anemia (FA) radial ray defects, we examined the patient-derived lymphoblastoid cells for molecular indicators of a defect in the FA pathway. We, therefore, measured frequencies of chromosome breakage following treatment with the DNA cross-linking agent 1,2:3,4-diepoxybutane (DEB), a robust assay employed clinically for the diagnosis of FA. However, we did not detect increased DEB-induced chromosome breakage in the TDP2 patient cells (Fig. 5A). In contrast, treatment with etoposide to specifically induce TOP2-induced DNA breaks did increase chromosome breakage in the TDP2^E152K^ cell lines significantly more than in the control cell lines (Fig. 5A, B). This further confirms the defect in repair of TOP2-induced DSBs in the TDP2-mutated patient cells. More importantly, whilst not as high as in TDP2 patient-derived cells, etoposide also induced high levels of chromosome breakage in the FA cell lines, when compared to control cells (Fig. 5A, B). This result suggests that the FA pathway, or at least components of the FA pathway, are required for the repair of TOP2-induced DNA damage. As discussed below, this observation may provide an explanation for the overlapping clinical pathologies in the current TDP2-mutated patients.

## Discussion

Spinocerebellar ataxia type 23 (SCAR23) (Gómez-Herreros et al. 2014; Zagnoli-Vieira et al. 2018; Ciaccio et al. 2019; Errichiello et al. 2020; Zoghi et al. 2021) is a very rare recessive neurological condition. Besides the progressive ataxia, affected individuals share other features including intellectual disability, microcephaly, developmental delay, and seizures (Table 1). In contrast to previously reported TDP2 patients, the current patients also exhibit short stature and skeletal abnormalities, including radial ray defects with hypoplastic thumbs, highly reminiscent of what is seen in Fanconi anemia. Exome sequencing in both siblings excluded additional mutations in one of the 23 known FA-related genes as a potential explanation. Despite this, TDP2 has not been reported to be a component of the FA pathway, and we did not detect any indications that this is the case. For example, we did not detect increased chromosome breakage following treatment with the DNA crosslinking agent DEB, in contrast to two FA patient cell lines employed in parallel. This is consistent with the reported specificity of TDP2 for repair of TOP2-induced DSBs, which reflects its ability to hydrolyze 5’-phosphotyrosyl bonds that link abortive TOP2 protein to the termini of such DSBs (Ledesma et al. 2009; Zeng et al. 2011; Schellenberg et al. 2012; Shi et al. 2012).

Alternatively, the FA-like phenotypes may reflect the nature of the novel *TDP2* mutation identified in our study. This mutation, E152K, is located within a highly conserved catalytic residue and inactivates the protein, as indicated by the absence of detectable 5′-tyrosyl-DNA phosphodiesterase activity in the patient cell extracts. Interestingly, a rare TDP2 SNP has been identified (D350N) that similarly resides in a residue critical for catalysis, though to date SCAR23 patients in which this allele is mutated have not been reported (Schellenberg et al. 2016). Critically, E152K results in reduced levels of cellular TDP2 protein. This is in contrast to all other TDP2 mutations currently reported in SCAR23, which greatly reduce or ablate TDP2 activity by affecting splicing or creating a premature stop codon and result in little or no residual TDP2 protein. It is thus tempting to speculate that the presence of residual but catalytically dead TDP2 confers additional, FA-like, clinical phenotypes that are not observed in TDP2 patients in which the mutated TDP2 protein is greatly reduced or absent. For example, it is possible that catalytically inactive TDP2 protein binds to and becomes ‘trapped’ on TOP2-induced DNA breaks, thereby preventing or impeding the compensatory repair of some of these lesions by other pathways, including the FA pathway. It is currently unclear what role the FA pathway might play in the repair of TOP2-induced DNA damage, since this DNA damage is comprised of protein-DNA crosslinks, whereas the canonical substrates for the FA pathway are DNA crosslinks (Peake and Noguchi 2022). Nevertheless, our observation that FANCC and F ANCG patient cells exhibit increased chromosome breakage following etoposide treatment is consistent with such a role. Indeed, hypersensitivity to etoposide has been observed in FANCD1/BRCA2 and FANCD2 defective cells, previously (Kachnic et al. 2011). Hypersensitivity of FA cells has also been reported to several other types of protein–DNA crosslinks (Elango et al. 2022).

Even though only a relatively small number of patients with SCAR23 have been reported, it is becoming clear that the phenotype of such patients can extend beyond neurological features and can include short stature, microcephaly, and subtle facial dysmorphic features. This observation reflects that the TDP2 protein is not only important for neural homeostasis but has a broader role in other tissues as well.
